# Sinus Venosus Atrial Septal Defect: A Challenging Diagnosis

**DOI:** 10.7759/cureus.5936

**Published:** 2019-10-17

**Authors:** Ziga Vodusek, Shehryar Khaliqdina, Carolina Borz-Baba, Rebecca Scandrett

**Affiliations:** 1 Internal Medicine, Frank H. Netter M.D. School of Medicine at Quinnipiac University, North Haven, USA; 2 Internal Medicine, Saint Mary's Hospital, Waterbury, USA; 3 Medicine, Saint Mary's Hospital, Waterbury, USA; 4 Cardiology, Saint Mary's Hospital, Waterbury, USA

**Keywords:** atrial septal defect (asd), atrial fibrillation, echocardiogram, trans-thoracic echocardiography, trans-esophageal echocardiography, syncope, anomalous pulmonary venous connection

## Abstract

Sinus venosus atrial septal defect (SVASD) is a rare adult congenital heart disease which permits shunting of blood from the systemic to the pulmonary circulation and is commonly associated with anomalous pulmonary venous return.

We report a case of a 27-year-old man with a history of premature birth and unilateral cryptorchidism who was admitted for syncope. Electrocardiogram (ECG) demonstrated atrial fibrillation (AF)and S1Q3T3 pattern along with an incomplete right bundle branch block. Transthoracic echocardiography (TTE) suggested the presence of right ventricular pressure and volume overload and severe right ventricular and right atrial enlargement. The agitated saline study was negative suggesting no inter-atrial communication. Transesophageal echocardiography (TEE) demonstrated a superior SVASD and raised the possibility of an anomalous pulmonary venous connection. Chest computed tomography identified the right superior pulmonary vein connection to the superior vena cava.

The diagnosis of SVASD poses multiple challenges from the variety of symptoms to the selection of appropriate imaging and the complexity of surgical treatment.

## Introduction

Unrepaired atrial septal defect (ASD) is the second most common adult congenital heart disease. Sinus venosus atrial septal defect (SVASD) is a non-primum, non-secundum variant, which represents only 5% to 10% of all types of ASD [1]. Atrial arrhythmias are commonly seen due to right heart volume overload secondary to the left-to-right shunt, but syncope is not among the typical initial clinical presentations of ASD [2]. Diagnosis of SVASD on transthoracic echocardiography (TTE) is particularly challenging and often necessitates the complementary use of more advanced studies, specifically transesophageal echocardiography (TEE), cardiac magnetic resonance imaging (CMR), cardiac computed tomography (cardiac CT), and rarely, cardiac catheterization [2]. Treatment is largely surgical and requires a thorough understanding of the associated complex anatomical abnormalities to guide an individualized surgical approach. 

We report a case of SVASD with an atypical presentation in a young patient with syncope in the setting of atrial fibrillation (AF), who had a negative TTE for an intracardiac shunt. 

## Case presentation

A 27-year-old man arrived at the emergency department (ED) after an episode of syncope. The patient was lifting off the floor a family member who had just experienced a fall, when he suddenly lost consciousness. He denied any presyncopal symptoms including nausea, vomiting, diaphoresis, dizziness, visual disturbances, chest pain, palpitations, abdominal discomfort, fever, and chills. Per his mother, who witnessed the event, the patient did not experience abnormal body movements or tongue biting or urinary incontinence during syncope. He was unconscious for several minutes but regained consciousness promptly and was not confused. After the syncopal event, the patient’s only complaint was tooth pain likely caused by local trauma during the fall. The patient did not recollect any head trauma and had no headaches prior to or after the incident. He had never had a similar event in the past.

The patient’s past medical and surgical history included unrepaired unilateral cryptorchidism and surgically corrected intussusception during infancy, both attributable to history of premature birth. He reported episodes of self-limited palpitations unrelated to exertion. He drank alcohol occasionally but had not had a drink for a week prior to admission. The patient never smoked or used drugs. He denied taking any medications or herbal supplements. He had no known family history of early-onset coronary artery disease, heart failure, valvular disease, arrhythmias, congenital heart disease, or sudden deaths. 

On physical examination, the patient appeared to be in no distress and was alert and oriented to person, place, time, and situation. Blood pressure was 140/90 mm Hg, pulse was 86 beats/minute (bpm), respirations were 12 breaths/minute, and the temperature was 97.8 °F. Weight was 106.6 kg and the body mass index was 39.11 kg/m². Orthostatic vital signs were not obtained upon arrival and were negative after fluid resuscitation received in the ED. The head was atraumatic. The neck was obese with normal carotid pulses and no bruits. Jugular venous distention and hepatojugular reflux were absent. The heart examination revealed an irregularly irregular rhythm and normal heart sounds. There was no right ventricular heave, murmur, nor friction rub noted. A right mid-abdominal well-healed scar was observed. There was no abdominal distention and the abdomen was non-tender. Bowel sounds were normal. Radial, dorsalis pedis, and posterior tibial pulses were normal bilaterally. 

Initial laboratory results revealed white blood cells 8.8 k/uL (normal range, 4.0-10.5), 79% (25% to 62%) of which were segmented neutrophils, hemoglobin 17.4 g/dL (12.5-16.0), platelets 329 K/uL (150-450), sodium 144 (136-145 mEq/l), potassium 4.5 (3.5-5.1 mEq/L), glucose 106 (70-105 mg/dL), troponin <0.03 ng/mL (<0.03), D-dimer ˂150 (0-230 DU ng/ml), creatinine 0.9 (0.7-1.3 mg/dl), blood urea nitrogen 14 (7-25 mg/dl), and TSH was 3.2 (0.45-5.33 uIU/mL). Urine toxicology was negative. 

The electrocardiogram (ECG) revealed atrial fibrillation (AF), with a ventricular rate of 83 bpm and an S1Q3T3 pattern along with an incomplete right bundle branch block. A repeat ECG completed four hours later demonstrated a return to normal sinus rhythm but with the persistence of the S1Q3T3 pattern and incomplete right bundle branch block. Broad differential diagnosis of right ventricular dysfunction in young adults was entertained, including pulmonary embolism, pulmonary hypertension from obstructive sleep apnea, congenital heart disease, and right ventricular dysplasia leading to ventricular arrhythmias. Computed tomography angiography (CTA) of the chest was performed in the ED. The test was negative for pulmonary embolism but described a 4.9-cm irregular low-density anterior mediastinal mass.

TTE revealed normal left ventricular ejection fraction estimated at 55% to 65% with abnormal septal motion suggestive of severely dilated right ventricle and right atrium. The left atrium had a normal cavity size with normal volume index. Rheumatic mitral valve disease with trace regurgitation and mild stenosis were noted. The tricuspid valve was thickened with trace regurgitation but no stenosis. Pulmonary artery systolic pressure was 24.4 mmHg, which was likely underestimated in the presence of trivial tricuspid regurgitation. Agitated saline contrast was given to evaluate for intracardiac shunt which was negative for the right to left anomalous inter-atrial connection. Unexplained right heart enlargement was further explored with TEE, which was performed the next day. 

**Figure 1:**
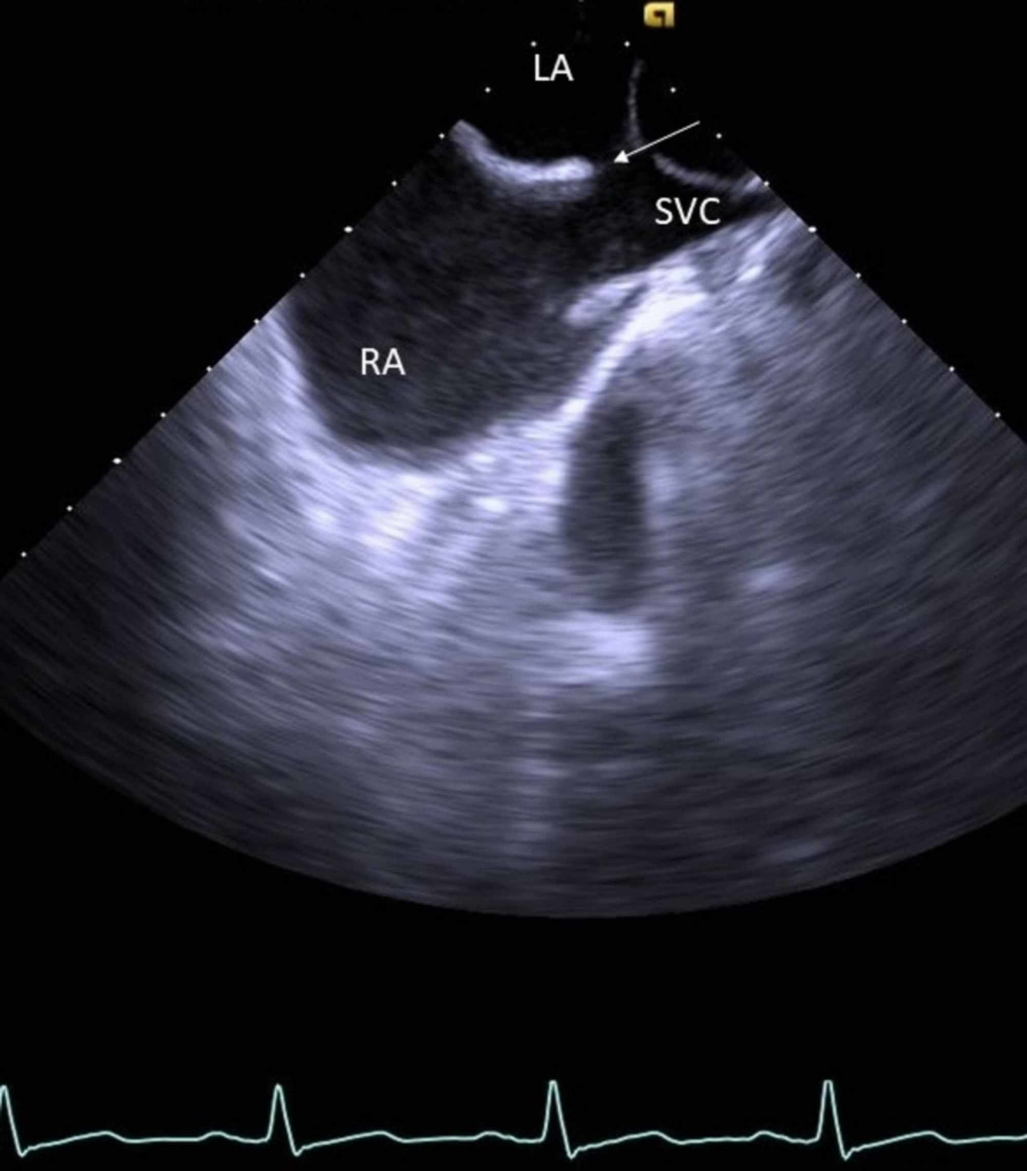
Transesophageal echocardiography The arrow shows superior sinus venosus atrial septal defect. LA, left atrium; SVC, superior vena cava; RA, right atrium

TEE revealed a large superior SVASD (arrow; Figure 1).

**Figure 2:**
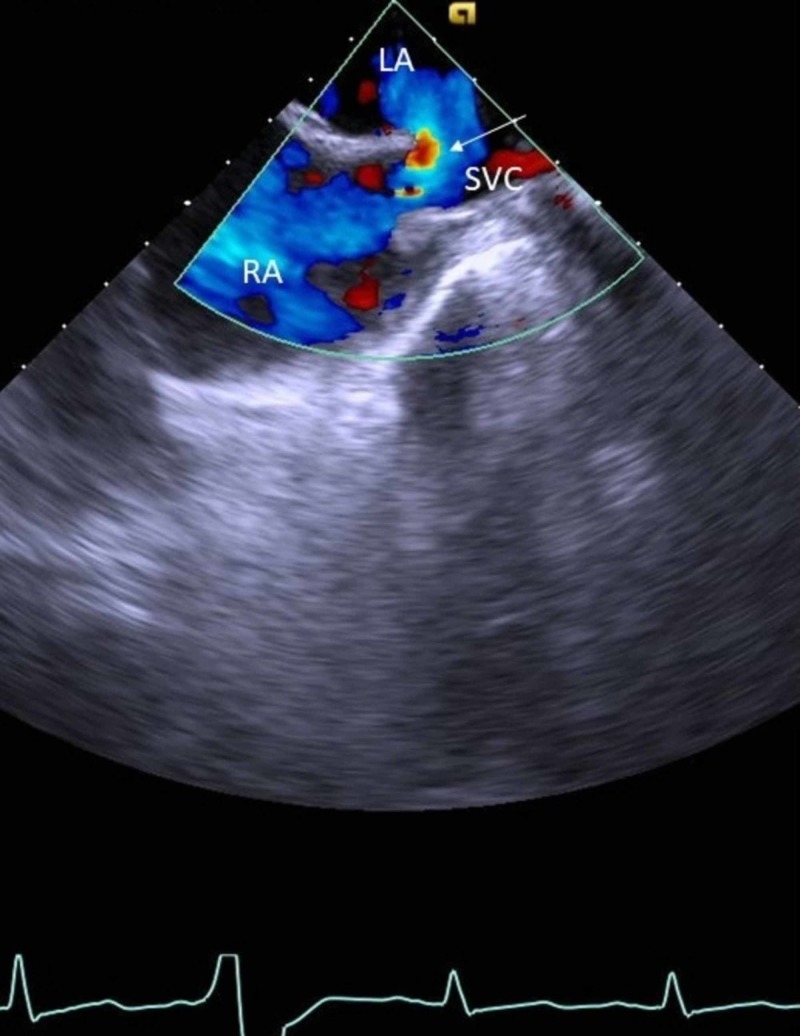
Transesophageal echocardiography with Doppler The arrow shows left-to-right shunting. LA, left atrium; SVC, superior vena cava; RA, right atrium

Left-to-right shunting (arrow) was indicated by color flow Doppler (Figure 2). 

**Figure 3:**
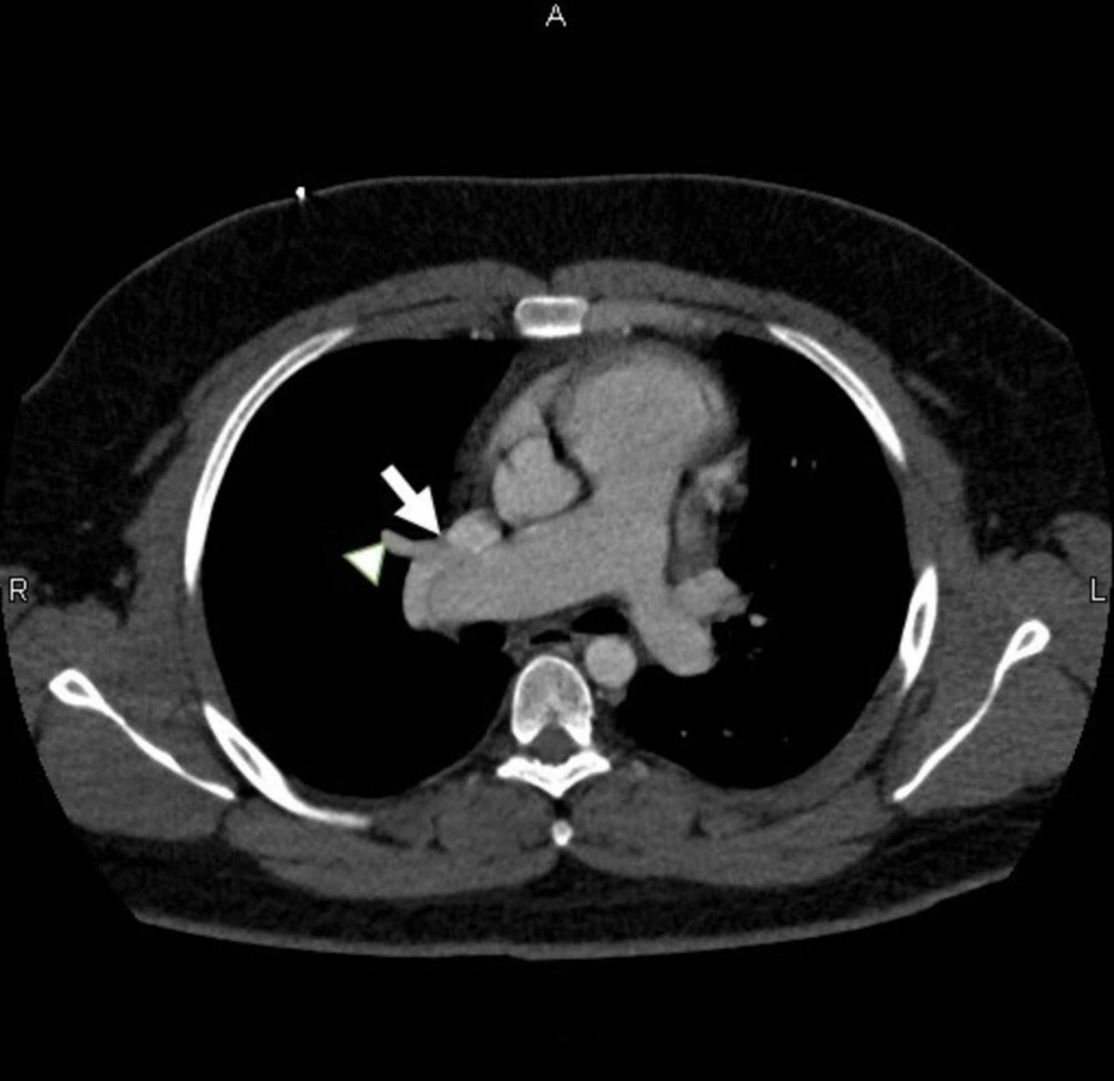
Computed tomography angiography Right superior pulmonary vein (arrowhead) connection to superior vena cava (arrow).

The CTA completed earlier (Figure 3) was reviewed with the radiologist who appreciated the right superior pulmonary vein connection to the superior vena cava (SVC). 

The patient was started on anticoagulation in the setting of AF with an ASD and was referred to cardiothoracic surgery. He underwent a successful ASD repair. During operation, it was confirmed that the right superior pulmonary vein drained into the SVC and it was noticed that the left superior vena cava crossed the hilum and joined the coronary sinus, which was dilated. The surgical technique performed was intra-atrial baffle repair of SVASD, which corrected the congenital defect. A patch method was used to augment SVC-right atrial confluence to achieve an unobstructed SVC connection to the right heart system.

During the procedure, the thymic mass was resected, and the pathology results were consistent with nodular lymphoid tissues with Hassall’s corpuscles and epithelial cells within a fatty stroma, recapitulating normal histology, consistent with hyperplasia. No atypia or malignancy was recognized. 

The patient made a good clinical recovery. The patient was discharged from the hospital five days postoperatively on oral aspirin 325 mg daily, metoprolol tartrate 50 mg twice daily and Tramadol 50 mg every six hours as needed for pain control. At the follow-up visit in the cardiology office, he reported very good exercise tolerance during daily activities and cardiac rehabilitation sessions.

## Discussion

SVASD is defined as an abnormal SVC connection with the superior rim of the atrial septum [3]. It is commonly associated with anomalous pulmonary venous return, which is a congenital abnormal connection of the pulmonary veins to the right atrium or to a systemic vein [4]. Left-to-right shunting leads to an overload of the right ventricle and pulmonary artery hypertension. Enlargement of the right ventricle impedes the diastolic filling of the left ventricle and subsequently increases the left-to-right shunt.

ASDs often resolve spontaneously during childhood. SVASD has a lower rate of spontaneous closure compared to other types of ASDs [5]. A recent study demonstrated that prematurity adversely affected spontaneous ASD closure [6]. Consistent with this observation, our patient was a young man with a history of prematurity, who also had unilateral cryptorchidism related to his preterm birth. 

The majority of patients with unrepaired large ASD (defined as greater than 10 mm) remain asymptomatic until the third decade of life [1]. The initial clinical presentation in adults includes dyspnea aggravated by exertion and palpitations [2]. Although supraventricular arrhythmia is a frequent complication of unrepaired ASD, syncope due to transient decrease in cardiac output leading to reduced cerebral perfusion is an infrequent initial clinical presentation of SVASD [1].

Physical examination findings of patients with ASD often include a wide fixed split-second heart sound, systolic ejection systolic murmur in the pulmonary valve area, and a precordial heave but these physical findings could be absent or limited due to body habitus [2].

ECG frequently depicts the right axis deviation, right bundle branch block, and inferior P-wave axis [4]. In our patient’s case, ECG demonstrated an S1Q3T3 pattern, an incomplete right bundle branch block, and AF with normal ventricular rate. The right ventricular dysfunction could be multifactorial in this case and associated with the presence of both unrepaired ASD and potentially undiagnosed obstructive sleep apnea.

Supraventricular arrhythmias are common in ASDs [7]. Among atrial tachyarrhythmias, the prevalence of AF is 10% to 15% in adults younger than 40-year-old, increasing up to 40% in adults 60 years of age [8]. The development of AF secondary to an ASD in young patients is related to the marked increase in pulmonary hypertension and subsequent atrial tissue remodeling [9]. Syncope and AF are entities that can precede each other but rarely occur in young patients without underlying cardiovascular disease. The simultaneous presentation of syncope, right ventricular dysfunction, and newly diagnosed AF in young adults, similar to our patient’s case, should prompt further cardiac testing to investigate adult congenital heart disease. 

TTE is the first-line diagnostic test in ASD. Prior studies have indicated that TTE was able to detect only 44% of SVASDs [10]. A recent retrospective analysis demonstrated that TEE provides better visualization of SVASD due to the proximity of the transducer to the defect [11]. TEE detects 20% of ASDs that were missed by the initial TTE [11]. In the same study, TEE identified all patients suspected of having SVASD based on TTE flow imaging or when performed for unexplained dilatation of the right side of the heart [11].

Adjunctive right heart echocardiographic data should be carefully evaluated in patients with SVASD, particularly right atrial and right ventricular enlargement, pulmonary artery pressure, and potential anomalous pulmonary venous connection [1]. In the presence of unexplained right atrial and ventricular enlargement, the possibility of underlying unrepaired ASD should be strongly considered [12]. 

Consistent with the echocardiography challenges in detecting SVASD reported in the literature, our patient’s TTE with agitated saline study did not detect septal abnormalities. It was not until TEE that a large SVASD was appreciated. 

Contrary to the other forms of ASD, the diagnosis of SVASD may require special cardiac imaging including CMR and cardiac CT to define the anatomical connection of pulmonary venous drainage into the RA [1]. In patients with known unrepaired ASD, the above-mentioned studies improve the detection of anomalous pulmonary venous connection and provide functional shunt parameters [13]. Diagnostic cardiac catheterization is rarely employed and is not advised in the perioperative evaluation of young patients with uncomplicated ASD [1]. 

Our patient also had mild rheumatic mitral valve stenosis (MS). The association between ASD and MS was first described by Lutembacher in 1916 [14]. This observation was demonstrated in other subsequent studies [9]. The association is known as Lutembacher syndrome. MS complicating non-primum atrial septal defects, like in our patient’s case is rare [15].

Management of SVASD includes medical treatment of the complications secondary to the presence of left to right shunt: heart failure, pulmonary hypertension, arrhythmias, angina. The surgical approach of SVASD is intricate and requires an individualized approach [16]. Repair of the SVASD has a good prognosis and is associated with low morbidity and mortality [16]. Patients with repaired SVASD have survival rates comparable with a matched population, and patients who underwent early surgical closure had better outcomes [16].

Postoperative complications in patients after SVASD closure are related to the type of surgery and atrial remodeling are more commonly described in older patients and include sinus node dysfunction and new-onset AF or flutter [16]. One particular challenge is represented by post-repair complications related to AF, particularly, if that was present before surgery, similar to our patient’s case. He returned to normal sinus rhythm shortly after the syncope and remained in normal sinus rhythm to date. The patient was not restarted on anticoagulation post-surgically. Anticoagulation after surgical closure remains an area of therapeutic uncertainty. Few case reports discuss stroke as a complication after SVASD surgical closure. A large retrospective study that followed patients who underwent ASD repair for a median of 16.8 years included 51 cases of SVASD [17]. The presence of AF before ASD closure increases the risk or recurrent AF post-repair especially in patients >40 years, and it seems to be the cause of stroke in 77% of patients after closure [17]. This suggests a potential benefit from closer monitoring or continuing anticoagulation postoperatively in patients who had AF before repair; however, there are no current guidelines favoring anticoagulation in these patients. Follow-up protocols including postsurgical periodic echocardiographic surveillance and cardiac monitoring may represent domains for future research.

## Conclusions

This case report highlights the importance of considering adult congenital heart disease in patients with syncope and unexplained right heart dysfunction. The diagnosis of SVASD can be very challenging in the presence of a negative TTE. Clinicians should carefully further assess these patients for potential rare variants of ASD and select additional appropriate cardiac imaging including TEE, cardiac CT, and CMR. An accurate diagnosis and timely referral for surgical repair prevents long-term sequelae from unrepaired SVASD and impact survival. 
